# Developing and evaluating a health pack to support dog owners to manage the weight of their companion animals

**DOI:** 10.3389/fvets.2024.1483130

**Published:** 2025-01-07

**Authors:** Thomas L. Webb, Jenifer Molina, Libby Sheridan, Hugues du Plessis, Joanna Brown, Hannah Abraham, Oliver Morton, Susan McKay

**Affiliations:** ^1^School of Psychology, The University of Sheffield, Sheffield, United Kingdom; ^2^Nestlé Purina PetCare, Barcelona, Spain; ^3^Mind Field Advertising, Manchester, United Kingdom; ^4^Perfect Circle, Manchester, United Kingdom; ^5^Companion Consultancy, Littleborough, United Kingdom

**Keywords:** obesity, overweight, weight management, dog, owner, behavioral science, COM-B model

## Abstract

**Introduction:**

Obesity is a serious and prevalent problem in dogs. The causes are multifactorial, but owners play a key role and so this paper reports the development and evaluation of a health pack designed to help owners to manage the weight of their dogs.

**Method:**

The pack was informed by previous research, behavior change theory (i.e., the COM-B model), and interviews with 12 veterinary professionals to identify challenges and potential solutions. Six workshops with a total of 28 dog owners provided feedback on the initial ideas. The pack included information on the importance of weight management, how to weigh and assess body condition score (BCS), a journal to track progress, an infographic illustrating the calorific value of treats, cards to help owners manage difficult situations, and a collar tag for the dog. The acceptability of the materials and potential outcomes were evaluated in a pre-registered pilot trial with a sample of 78 dog owners who were posted a health pack, 49 of whom completed a follow-up questionnaire.

**Results:**

The findings suggested that owners were willing to weigh their dog, found the pack acceptable, and there was preliminary evidence that the weight and BCS of dogs was lower at follow-up than at baseline.

**Discussion:**

The findings illustrate the potential of a health pack for supporting dog owners and provide the basis for a larger RCT to formally evaluate effectiveness.

## 1 Introduction

Obesity among companion animals is a serious and increasingly common problem. The prevalence of canine obesity ranges between 25 and 44% in developed countries (1) and, according to the results of the Pet Obesity Survey (2), the prevalence of obesity is increasing. Obesity is a medical condition characterized by the accumulation of excess body fat to a degree that can adversely impact health (3). Rather than being a passive tissue, excessive body fat is recognized to have far-reaching effects on various organ systems through inflammatory, neuroendocrine, and other mechanisms. Overweight dogs are particularly susceptible to a range of health issues, including metabolic, endocrine, renal, urinary, respiratory, orthopaedic, dermatological, neoplastic, and ophthalmologic conditions (4–7). These animals often experience a diminished quality of life (8–10) and have shorter lifespans, with evidence that obese dogs die on average 1–2 years earlier than their non-obese counterparts (11–12). Therefore, identifying ways to detect, prevent, and manage obesity in companion animals is critical.

Risk factors for obesity among companion dogs are multifactorial and include factors pertaining to the dog (e.g., genetics, breed, neutered status, age, sex, responsiveness to food, and diseases like diabetes mellitus or hypothyroidism) (7, 13, 14). However, owners typically control food intake and can moderate energy expenditure among companion animals, which has led some to advocate for a behavioral science approach to managing obesity among companion animals that focuses on the owner, rather than – or in addition to – their dog (15, 16). A number of factors related to the owner have been shown to be associated with overfeeding and poor weight outcomes, including not realising that the dog is overweight (17, 18), not appreciating the risks of obesity (19) or associating costs with dog ownership (20), owners’ levels of physical (in)activity (21), using food as a way to bond with the dog (22), allowing the dog to be present when preparing or eating food (23) and excessive use of treats (24, 25).

While research is making progress identifying the factors that influence the way that owners feed and exercise their companion animals, less research has translated these insights into practical and acceptable interventions that can support owners to make changes to their behavior. There are examples of successful weight loss programmes, but these typically focus primarily on caloric restriction or other dietary changes and are implemented in controlled environments (26–29). Outside such controlled environments, veterinary professionals try to advise and support owners [e.g., via nurse-led clinics (30, 31)]. However, there is limited time in such consultations, veterinary professionals may find discussing obesity challenging (32–35), owners may dismiss or struggle to follow advice (36), and recommendations may not translate into the owner’s home environment (37). Indeed, interventions delivered outside the laboratory are often associated with relatively low levels of compliance (38, 39). For example, German et al. (40) found that only around 60% of dogs referred to a controlled feeding programme completed the programme. Reasons for stopping prematurely included refusal to comply with weight management advice.

Taken together, there is a clear need for an intervention that can support owners to make changes to their behavior - and perhaps support veterinary professionals to work with owners to do so. Interventions have targeted owners’ behavior. For example, a review by Krasuska and Webb (41) identified 14 interventions targeting owners’ behavior. However, with a few notable exceptions that examined the effect of monthly classes on nutrition (42) and collaborative exercise programs (43–45), the majority of the studies simply recommended that owners feed less or differently but provided little or no support for so doing (46). Other interventions required access to specialist equipment [e.g., an underwater treadmill (47)]. Webb et al. (48) tried to support owners by developing a volitional help sheet (49) designed to help owners to form if-then plans specifying how to deal with challenging situations (e.g., ‘If my dog is begging for a treat, then I will give him/her a cuddle instead!’). Over 600 studies support the idea that this type of planning [known as ‘implementation intentions’ (50)] helps people to achieve their goals (51); yet Webb et al. (48) found no difference in the behavior of owners in the intervention condition and those in the control condition who did not receive the help sheet. One reason was that the helpsheet was embedded at the end of a relatively long questionnaire and less than half of the sample completed the planning exercise.

This example points to the importance of ensuring that interventions are acceptable to those who are expected to engage with and use them. Acceptability refers to the extent to which people receiving the intervention consider it to be appropriate, based on anticipated or experienced cognitive and emotional responses to the intervention (52). Acceptability has become a key consideration in the design, evaluation and implementation of healthcare interventions (52), but seems to be rarely considered in preventive veterinary medicine, despite evidence that compliance with advice and interventions is often low (38–40) and that owners may not believe that recommendations are acceptable. For example, MacMartin et al. (36) reported that owners found nutritional suggestions made by veterinarians potentially unnecessary, inappropriate, or unfeasible. Acceptability is a necessary precondition for an intervention to be effective and the reason why those developing interventions – including the present team – view an ‘acceptability trial’ as critical. We need to know that owners are willing to use an intervention before we can assess whether the components of the intervention influence outcomes.

Evidence suggests that interventions that are based on theory are typically more effective than those that are not and theory can provide a framework for developing interventions (53). The COM-B model developed by Michie et al. (54) is one of the most influential frameworks for understanding behavior and developing interventions. The model suggests that three components are needed for any behavior to occur. First, the person must be motivated; that is, they must want, or appreciate the need, to behave in a certain way (e.g., take steps to change the way that they feed their dog because they recognise that their dog is overweight and appreciate the risks of being overweight). However, the COM-B model recognises that motivation alone is unlikely to lead to changes in behavior [cf. research on the gap between intentions and behavior (55–56)] and therefore proposes that the person must also be capable and have the opportunity to act. Capability refers to whether the person has the physical strength, knowledge, skills, stamina etc. to perform the behavior (e.g., an owner who does not know when, what, or how much to feed their dog is unlikely to be able to feed appropriately (48)). Opportunity reflects the need for a conducive physical and social environment for behavior (e.g., an owner needs a measuring cup or small set of scales in order to feed an appropriate amount of food). The COM-B model has been used to develop a wide range of interventions, including in veterinary science [e.g., to understand behaviors such as disease control among cattle farmers (57–59) and obesity among horses (60)]. It therefore has the potential to provide a useful framework for understanding dog owners’ behavior and informing the design of an intervention designed to support changes where needed.

The aim of the proposed research was to develop a health package that will support owners to change their behavior and reduce weight among companion animals. The preceding discussion suggests that interventions need to draw on theoretical frameworks and insights from behavioral science - yet also be developed alongside owners to ensure that they are accessible and acceptable. The research therefore involved two phases – (i) a development phase and (ii) evaluation of acceptability and preliminary evaluation of effects on potential outcomes, including owners’ behavior and weight of their companion animals.

## 2 Phase 1: development

Understanding from current evidence (e.g., that reviewed in the introduction) was supplemented with interviews with veterinary professionals to identify key issues that owners encounter managing the weight of their dogs and potential strategies for supporting owners. This information was then used to compile an initial set of tools for the health pack. These initial tools were mapped to COM-B components to ensure that they targeted all of the theoretical determinants of behavior (i.e., capability, opportunity, and motivation) and were then presented to owners via a series of collaborative workshops to obtain feedback. The tools were then refined with the support of a marketing agency to create the final health pack.

### 2.1 Interviews with veterinary professionals

#### 2.1.1 Materials and method

**Figure 1:**
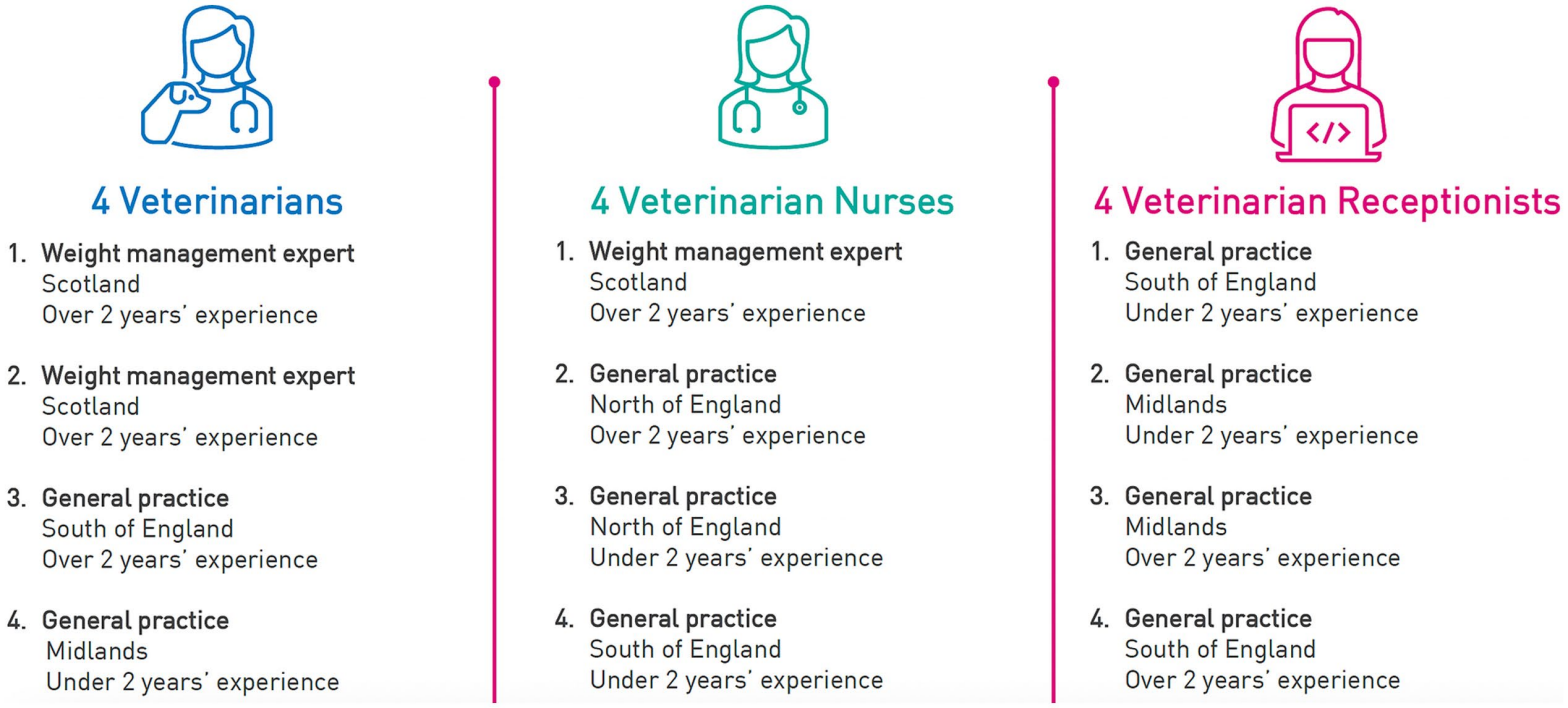
Characteristics of the veterinary professionals who took part in Phase 1.

Semi-structured interviews were conducted with 12 veterinary professionals (4 veterinarians, 4 veterinary nurses, and 4 veterinary receptionists^1^) who worked at or owned a general veterinary practice that serves dogs. The professionals were recruited by the 5th author from a database held by Central Fieldwork and were selected to represent a range of geographical regions in the UK and experience – in veterinary practice and in weight management. Figure 1 shows the characteristics of the participants in this phase of the research.

Each interview focused on the professionals’ experience of how owners manage obesity, including the issues that owners face and the strategies that the owners and veterinary practices employ to address these issues (see Supplementary Material 14.1 for the full interview guide). The interviews were recorded and analyzed to identify common themes, which were categorized into those relating to motivation, capability and / or opportunity using the COM-B model as a framework.

#### 2.1.2 Results

The professionals identified a range of reasons that they believed led dogs to become overweight (see Table 1). The issues reflected all of the COM-B components including psychological capability (e.g., knowing how to monitor weight and intake), physical capability (e.g., the owner’s own capacity to exercise), social opportunity (e.g., family norms around feeding), physical opportunity (e.g., reduced engagement with veterinary professionals), reflective motivation (e.g., not giving sufficient attention to preventive care), and automatic motivation (e.g., humanization of companion animals).

**Table 1:** Issues and strategies identified by veterinary professionals for managing obesity, categorized by COM-B component.

COM-B Component	Issues identified by professionals	Potential strategies identified by professionals
Psychological capability	Not knowing how to weigh a dog. Not knowing how to weigh food. Not monitoring intake. Information on the packaging of food is unclear, hard to follow, and / or not tailored to the breed and size of the dog. Lack of experience with ownership (e.g., dog purchased on impulse).	Weighing out daily allowance for the day in the morning and only feeding from that allowance. Weighing the correct amount of food once and marking on a cup where that food stops so as to not over fill in future. A step-by-step journal and weight loss progress chart could provide a visual tool. Receptionists sell the recommended food and so can give advice on how to read on pack instructions for amounts and frequency.
Physical capability	Difficulty weighing larger breeds. Owners’ own capacity to exercise is limited.	Provide a guide on how to weigh.
Social opportunity	Vets are sometimes uncomfortable addressing weight problems Formal and informal social norms: visuals of dogs on social media and in the community. Family norms (i.e., that dogs should be given leftovers rather than them going to waste)	Contact owners via Zoom Involve more of the family in looking after the dog and discovering new feeding and exercising tips.
Physical opportunity	Cost of going to the vets may be prohibitive. Engagement with vets has decreased during the COVID-19 lockdown. Increased working from home (e.g., as a result of the COVID-19 pandemic)	Receptionists can offer advice and signpost to online resources for clients who worry about costs. A visual to illustrate the cost of obesity (e.g., a ‘cost calculator’) could help conversations about cost of treatments. Switching to low calorie food instead of the normal type.
Reflective motivation	Treats being seen as a normal part of having a dog (i.e., used to show love, to train and to build trust between animal and humans). Owners do not see quick, positive results and so give up. Owners do not see the problem and / or are not ready to address it. Owners rarely consider preventive care, only dealing with weight once it becomes a problem.	Leverage owners tendency to humanize their dog, by humanising the treats that they give them (i.e., show what they would be equivalent to a human eating). Buy low calorie treats and ensure that they are calculated with meals within the daily calorie amount. Give vegetables as healthy treats (e.g., carrot sticks). Additional distraction ideas: a belly rub, learn a new trick, go for a walk, give a toy, throw a ball. Being present in the clinic is a strong motivator. Posters in the waiting room can encourage owners to start thinking about weight problems. This helps open up the conversation with owners and demonstrates the practice’s advice. Having a physical product to hand out in practice could help to keep owners motivated and reinforce the verbal advice given by the vet. The best motivation is seeing results.
Automatic motivation	Humanization (e.g., professionals felt that owners often viewed exercise in human terms - not appreciating how much exercise dogs need).	A story book could help educate the next generation of dog owners. Positive language that allows children to understand what a healthy dog looks like.

The research team then used the feedback from the professionals alongside insights from research on obesity among companion animals to develop an initial set of tools to support owners (see Table 2 for a summary). These tools were designed to target each of the determinants of behavior specified by the COM-B model (i.e., capability, opportunity, and motivation) and described in terms of the behavior change techniques [the smallest parts of an intervention that are observable, replicable, and on their own have the potential to bring about behavior change (54)] that they employed (specified using the Behavior Change Technique Ontology (61)). Using standardized frameworks to describe the content of behavioral interventions provides transparency, allows replication, and the accumulation of evidence about the effectiveness of interventions (68).

**Table 2:** Summary of the initial set of tools to support dog owners.

Tool	Brief description See the concept boards on the open science framework (https://doi.org/10.17605/OSF.IO/D9ZTP) for more detail	Targeted COM-B process(es)	Behaviour Change Techniques (BCTs), specified using the Behaviour Change Intervention Ontology [BCIO, Marques et al. (1)]
Guide on how to use the pack	Booklet providing an overview of the tools in the pack, how and why these have been created and how to use them.	Psychological capability Reflective motivation	BCIO:007001: Goal-directed BCT BCIO:007050: Guide how to perform behavior BCIO:007075: Present information from a credible source
How to weigh and score BCS	Instructions on how to weigh a dog (e.g., standing on scales holding dog) along with tool for scoring body condition (BCS)	Psychological capability Physical capability	BCIO:007058: Instruct how to perform a behavior BCIO:007301: Set measurable outcome goal
Journal for tracking progress	A booklet intended to help owners to keep track of the dates of weigh ins, changes to diet, exercise, plan meals.	Psychological capability Reflective motivation	BCIO:007300: Set measurable behavior goal BCIO:007024: Self-monitor behavior BCIO:007010: Action planning
Weight loss progress chart	A chart linked to the journal which can be used to monitor weight. Rewards (e.g., stickers, magnets) can be placed over dates where weight is lost.	Psychological Capability	BCIO:007025: Self-monitor outcome of behavior
Roles and responsibilities planner	A wipeable wall chart, linked to the journal to plot householders’ roles and responsibilities with respect to their pet (e.g., who will play, feed dinner).	Psychological capability Social Opportunity	BCIO:007024: Self-monitor behavior BCIO:007028: Social support
Treat calculator - infographic	An infographic which illustrates the calorific value of treats by showing the human equivalent (e.g., that 1 rawhide bone to a dog is equal to a human consuming 7 doughnuts).	Psychological capability Reflective motivation	BCIO:007062: Increase awareness of consequences BCIO:007173: Increase awareness of behavior BCIO:007302: Suggest different perspective on behavior
Treat calculator - card game	A card game that builds on the infographic to allow players to match dog treats with their human equivalent.	Psychological capability Reflective motivation	BCIO:007062: Increase awareness of consequences BCIO:007173: Increase awareness of behavior BCIO:007302: Suggest different perspective on behavior
Story book	Illustrated story book intended to engage children. Tells the tale of a dog that is loved by a family, but is fed too many treats, which makes him unhappy and overweight. The family then change their feeding and exercise habits making the dog happy again.	Reflective motivation	BCIO:007062: Increase awareness of consequences BCIO:007179: Inform about negative health consequences
Dog obesity cost calculator	Illustration of a dog, with annotations demonstrating different health conditions relating to obesity along with the potential cost (e.g., average medical bill at the vets) for each.	Reflective motivation	BCIO:007062: Increase awareness of consequences
Scales to weigh food	Small set of scales to help owners weigh out the appropriate amount of food.	Physical opportunity	BCIO:007163: Add objects to the directly experienced environment
Slow feeder	A plastic mat, divided into segments that can be placed at the bottom of a standard dog bowl to slow down eating.	Physical opportunity	BCIO:007163: Add objects to the directly experienced environment
Collar tag (“I’m on a special diet”)	Badge / tag to be attached to the dog’s collar. Serves as a visual reminder to avoid excessive feeding.	Social opportunity Automatic motivation	BCIO:007081: Cue BCIO:007163: Add objects to the directly experienced environment
If this then that flip cards	A form of volitional help sheet (48, 49), which allows owners to make if-then plans specifying how to deal with potential challenges (e.g., If I’m tempted to share what I am eating with my pet because they beg for food, then I will remind myself that my dog just wants my attention and play instead!)	Psychological Capability	BCIO:007010: Action planning
Action /distraction	A tool to help owners identify an alternative activity (e.g., throw ball, rub belly) if the dog is begging for food. Owner rolls a dice and then looks up the number on a wheel which suggests activity.	Psychological capability	BCIO:007095: Substitute behavior BCIO:007143: Enable person to manage automatic responses BCIO:007154: Advise distraction BCIO:007163: Add objects to the directly experienced environment BCIO:007303: Suggest how to perform behavior

### 2.2 Collaborative workshops with dog owners

#### 2.2.1 Materials and methods

Feedback on the initial set of tools was obtained from dog owners via a series of collaborative workshops. Ethical approval was granted by the Research Ethics Committee at the University of Sheffield (application #044619). Participants were recruited by the 5^th^ author from a database held by Central Fieldwork. Potential participants were asked to complete a screening questionnaire from which owners who described having at least one dog that they deemed overweight were identified.^2^

Six workshops were conducted with 28 dog owners (9 male and 19 female) in three cities in the UK (Leeds, Nottingham, and Watford) in February 2022. Four of the owners (14%) did not have children, 18 (64%) had children living at home, and 6 owners (21%) had children who had left home. In terms of ethnicity, the majority of the sample (26 participants, 93%) were White British. Seventeen of the participants (61%) had 1 dog in the home and 11 of the participants (39%) had 2 or more dogs. Eleven of the participants (39%) had a small dog, 12 (43%) had a medium-sized dog and 5 (18%) had a large breed of dog. Sixteen of the participants (57%) described their dog as overweight, 2 (7%) as quite overweight and 10 (36%) as very overweight.

On arrival at the workshop, participants received information about the research, provided informed consent and were told that they would receive £70 for taking part. Each workshop lasted around 90 min and involved participants introducing themselves, saying a bit about their dog, as well as whether and how they manage the weight of their dog. The main part of the workshop involved showing participants visuals of the initial set of tools for the health pack on large ‘concept’ boards (these can be viewed on the Open Science Framework https://doi.org/10.17605/OSF.IO/D9ZTP). The moderator introduced each board and then asked participants to walk around (in pairs or small groups if wanted) and use Post-it notes to indicate (i) what they liked and why (green Post-it notes), (ii) what they did not like and why (red Post-it notes), and (iii) how they would improve it (yellow Post-it notes). Participants were then asked to share their feedback with the group and to discuss and decide which tools they felt would be most effective in helping owners to manage their dog’s weight. Finally, participants were asked to think about their personal circumstances (e.g., lifestyle, work pattern, household members) and the needs of their dog in particular and to use purple Post-it notes to identify (i) which of the tools they would see being used in their household, (ii) how they would be used and by who, and (iii) how they would help to change what they do now.

#### 2.2.2 Results

Table 3 provides a summary of owners’ feedback on the initial set of tools. This feedback was used in a series of meetings with a marketing agency to develop and refine the tools and decide which to take forward into the final pack. It was decided to retain the guide on how to use the pack (split into a cover letter and booklet), how to weigh and score BCS, the journal for tracking progress, the roles and responsibilities planner (which became a box prompting owners to reflect on ‘who’s in charge’ of each of the three behaviors specified in the journal for tracking progress), the weight loss progress chart (which became referred to as the ‘weekly weight tracker’), the treat calculator infographic, collar tag, and the action / distraction tool in the form of ‘healthy weight care cards’. We removed the treat calculator card game, story book, dog obesity cost calculator, scales to weigh food, slow feeder, and the ‘if this, then that’ flip cards. We also added learning points (explaining why weight management is important) and a monthly milestones chart (in addition to weekly monitoring of weight) that included goal setting and review.

**Table 3:** Summary of owners’ feedback on the initial set of tools.

Tool	Feedback and suggestions
Guide on how to use the pack	A guide to how to use the pack was deemed essential to explain how each tool works, and why it has been included. However, owners felt that some degree of personalization would be helpful to work for different dogs and households.
How to weigh and score BCS	Owner’s felt that information on how to weigh and assess BCS would be essential for new owners. Noted that local pet stores often have scales for weighing dogs.
Journal for tracking progress	Deemed useful, but potentially quite time consuming. Suggested that could be more interactive.
Weight loss progress chart	Owners like the idea of linking progress to rewards - e.g., “*This would be good for us as we are quite organised as a family. The reward would have to be something for everyone like a trip to the park or seaside*.” Some concern about the focus on weight loss being stigmatising - e.g., “*My kids are 9 and 12 and I do not want them thinking about weight loss. They just know about eating healthy and exercising*.”
Roles and responsibilities planner	Owners stated that could be used to motivate children to get involved: “*At the end of the week if Sam has gone out with Dad twice to walk the dog and given him breakfast 3 times he gets a day out in the trampoline park / a fiver*.”
Treat calculator - infographic	Owners expressed surprise by this information and suggested that it would be useful - e.g., “*I know ham is bad, but then he looks at me with those eyes and I cave and go to the fridge. A reminder of how bad ham is on the fridge is exactly what I need*.”
Treat calculator - card game	Families like the concept, but felt that it would be played once or twice and then put in a drawer.
Story book	Owners suggested a positive framing to avoid ‘diet culture’ stigma. Owners also suggested incorporating adult humor like Horrible Histories and Wonky Donkey to engage parents when reading to children.
Dog obesity cost calculator	Owners reported that could motivate new dog owners - e.g., “*If you do not overfeed…you will save £x per year*.” Families reported that the cost felt insignificant compared to the cost of having children.
Scales to weigh food	Owners preferred measuring cups to scales.
Slow feeder	“*I do not understand how that helps them lose weight…they are eating the same amount of food!*”
Collar tag (“I’m on a special diet”)	Owners reported that “I’m on a special diet” works well because it could relate to health so people are more likely to abide by it. Owners felt that the tag could easily be ignored on the collar and potentially cause stigmatization.
If this then that flip cards	Owner’s suggested that the cards should give examples of activities. Some owners felt that action planning may need to be supported by a professional.
Action /distraction	“*It would work great for me when I’m cooking dinner and the kids are running riot and the dog is pestering for food – I can distract them both by giving them the dice*” “Go for a walk” or “Learn a new trick” was considered off-putting and not practical (e.g., when in the middle of cooking a meal).

Table 4 provides an overview of the final set of intervention components alongside (i) the component of the COM-B model that they target and (ii) the Behavior Change Techniques [BCTs (61)] that they use to do so, specified using the Behavior Change Intervention Ontology [BCIO (62)].

**Table 4:** Overview of the intervention components.

Intervention component	Targeted COM-B component	Behavior Change Techniques (BCTs), specified using the Behavior Change Intervention Ontology [BCIO, Marques et al. (1)]
Cover letter and guide on how to use the pack	Psychological capability Reflective motivation	BCIO:007001: Goal-directed BCT BCIO:007050: Guide how to perform behavior BCIO:007075: Present information from credible source BCIO:007206: Promise behavioral consequence for completion of a behavioral sequence
Main booklet		
“Learning points” - Why is weight management important?	Psychological capability Reflective motivation	BCIO:007062: Increase awareness of consequences BCIO:007173: Increase awareness of behavior BCIO:007179: Inform about negative health consequences
Treat calculator - infographic	Psychological capability Reflective motivation	BCIO:007062: Increase awareness of consequences BCIO:007173: Increase awareness of behavior BCIO:007302: Suggest different perspective on behavior
How to weigh and score BCS	Psychological capability Physical capability	BCIO:007058: Instruct how to perform a behavior BCIO:007301: Set measurable outcome goal
Journal for tracking progress - ‘12 weeks to healthier habits’ includes daily monitoring of three behaviors (measure food, extra exercise and playtime, replace snacks and treats with healthier options)	Psychological capability Reflective motivation	BCIO:007300: Set measurable behavior goal BCIO:007024: Self-monitor behavior
Roles and responsibilities planner - journal above includes box to specify ‘Who’s in charge?’ of each behavior	Psychological capability Social opportunity	BCIO:007024: Self-monitor behavior BCIO:007028: Social support
Weight loss progress chart	Psychological capability	BCIO:007025: Self-monitor outcome of behavior
Monthly milestones chart, including goal setting and review	Psychological capability Reflective motivation	BCIO:007012: Attend to discrepancy between current behavior and goal BCIO:007013: Review outcome goal BCIO:007025: Self-monitor outcome of behavior BCIO:007301: Set measurable outcome goal
Additional tools		
Healthy weight care cards	Psychological capability	BCIO:007010: Action planning BCIO:007095: Substitute behavior BCIO:007143: Enable person to manage automatic responses BCIO:007303: Suggest how to perform behavior
Collar tag (“I’m on a special diet”)	Social opportunity Automatic motivation	BCIO:007081: Cue BCIO:007163: Add objects to the directly experienced environment

## 3 Phase 2: evaluation

The second phase of the research sought to evaluate the acceptability of the health pack and its potential to support owners. This research should therefore be considered the ‘pilot or feasibility phase’ ahead of the ‘evaluation’ and ‘implementation’ phases (52) and was not intended to evaluate whether receipt / use of the pack leads to changes in behavior and / or outcomes for the companion animal (although we measured these things, the study did not randomise participants to condition, nor did it have sufficient statistical power to detect changes over time). The evaluation of the health pack and hypotheses below were pre-registered on the Open Science Framework (https://osf.io/e65wj).

### 3.1 Hypotheses

Primary hypotheses.

1. Owners will be willing and able to self-assess and report their dog’s weight^3^ and Body Condition Score (BCS).
2. Owners who are posted a health pack will report using that pack.
3. Owners will find the health pack acceptable, as evidenced by agreement with items reflecting (i) affective attitudes, (ii) perceived effectiveness, (iii) intervention coherence, and (iv) self-efficacy, and disagreement with items reflecting (i) burden, (ii) ethicality, and (iii) opportunity costs.

Secondary hypotheses.

4. Owners who are posted a health pack will report (i) changing the way that they feed, exercise, or interact with their dog over the 8 weeks following receipt of the pack, and (ii) these changes will be the result of receiving or using the health pack.
5. The weight and BCS of dogs will be lower at follow-up than at baseline.

### 3.2 Materials and method

Ethical approval was granted by the Research Ethics Committee at the University of Sheffield (application #055570) and the internal ethics committee at Purina PetCare.

#### 3.2.1 Participants

**Figure 2:**
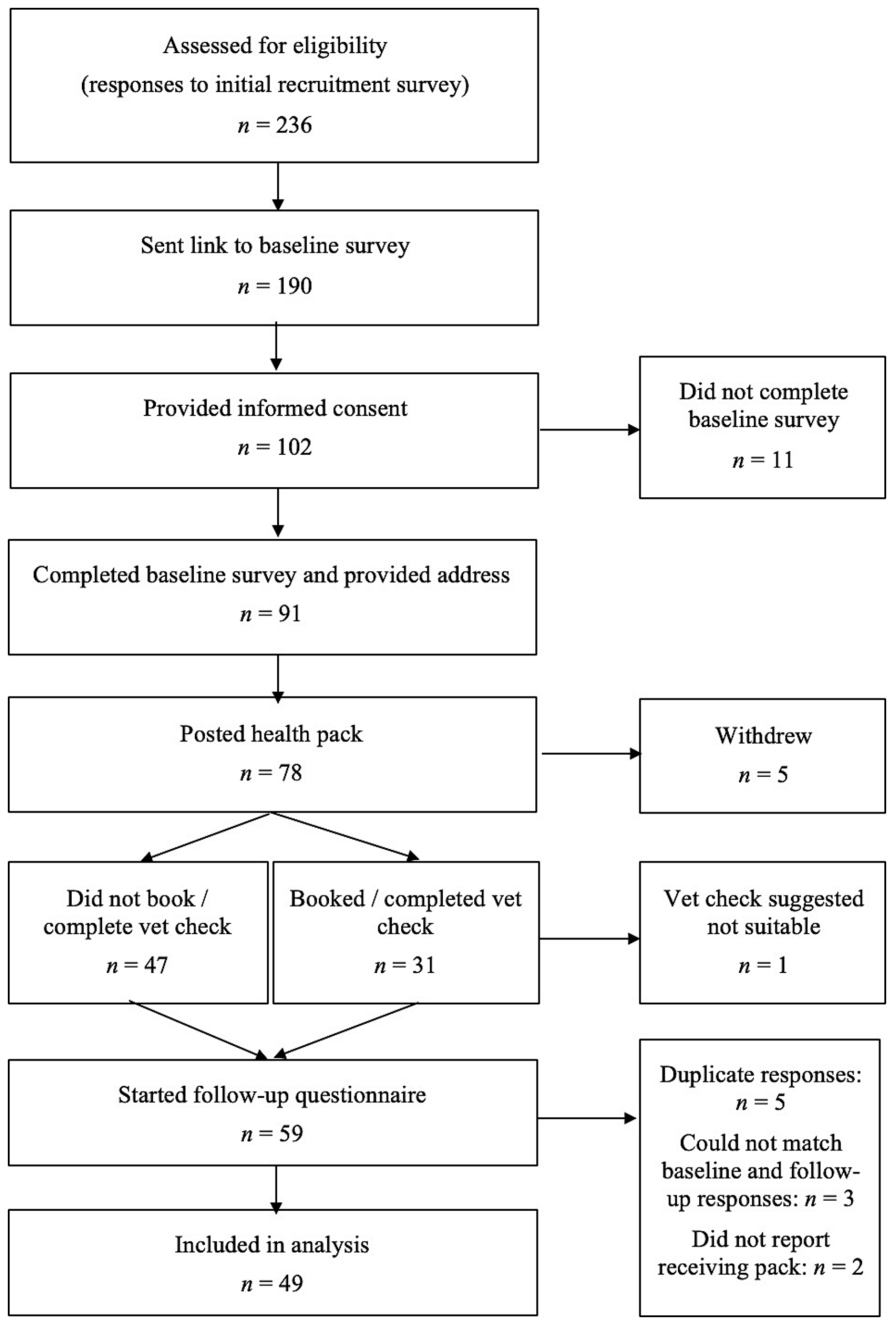
CONSORT diagram showing the flow of participants through the evaluation study.

There are few criteria for identifying the sample size needed to investigate the acceptability of an intervention and previous studies with relatively small samples have been highly cited. For example, Ben-Zeev et al. (63) recruited 33 participants to assess the acceptability of a smartphone intervention for schizophrenia. We therefore decided to aim for 50 participants with complete data (i.e., completed pre- and follow-up measures) and to recruit up to 80 participants, assuming that not all would complete the follow-up measures. Owners of overweight dogs were recruited using a snowball recruitment method, starting with owners in the UK that were known to the recruitment agency and via an advert posted on Facebook, Instagram and LinkedIn and in local places across the UK. The sample did not include owners who had taken part in the research used to develop the pack. Owners were not incentivized to complete the initial questionnaire or use the toolkit, but were told that they would receive a small incentive (£25) for answering the second questionnaire. Figure 2 shows the flow of participants through the study and Table 5 shows the characteristics of participants at baseline.

**Table 5:** Characteristics of dogs and owners at baseline.

	All participants who completed the baseline survey (*N* = 91)	Analyzed sample (i.e., those that completed follow-up survey and were matched to baseline) (*N* = 49)
Age (participant)	*M* = 40 (*SD* = 9)^1^	*M* = 40 (*SD* = 10)
Gender (participant)		
Female Male	90.1% 9.9%	87.8% 12.2%
Employment status		
Full time Part-time Unemployed Retired Other	68.1% 14.3% 4.4% 1.1% 12.1%	69.4% 12.2% 4.1% 2.0% 12.2%
Type of residence		
Flat Terraced house Semi-detached house Detached house Other	8.8% 24.2% 42.9% 23.1% 1.1%	6.1% 22.4% 46.9% 24.5% 0.0%
Number of adults in household	*M* = 2.15 (*SD* = 0.68)	*M* = 2.18 (*SD* = 0.78)
Number of children in household	*M* = 0.35 (*SD* = 0.64)	*M* = 0.37 (*SD* = 0.67)
Number of dogs	*M* = 1.57 (*SD* = 1.01)	*M* = 1.57 (*SD* = 1.15)
Is this the first dog that you have owned?		
Yes No	38.5% 61.5%	38.8% 61.2%
Are you primarily responsible for the care of your dog?		
Yes Share equally	86.8% 13.2%	89.8% 10.2%
Age (dog)	*M* = 4.88 (*SD* = 2.80)	*M* = 4.45 (*SD* = 3.00)
Gender (dog)		
Male Female	49.5% 50.5%	57.1% 42.9%
Breed	Wide range	Wide range
What size is your dog?		
Toy Small Medium Large	1.1% 30.4% 50.0% 18.5%	0.0% 42.9% 40.8% 16.3%
Is your dog neutered?		
Yes No	78.0% 22.0%	81.6% 18.4%
Has your dog been diagnosed with a chronic or terminal illness?		
Yes No	6.6% 93.4%	4.1% 95.9%
Weight (kgs)	*M* = 19.10 (*SD* = 11.44)	*M* = 17.86 (*SD* = 12.20)
Body condition score (9 pt. scale)	*M* = 5.78 (*SD* = 1.11)	*M* = 5.69 (*SD* = 1.12)

Note that participants’ age is reported to the nearest whole year as estimated by subtracting year born from year completed survey.

*N, number of participants; M, mean, SD, standard deviation.*

#### 3.2.2 Procedure

##### 3.2.2.1 Initial questionnaire

Owners were contacted by email and asked to complete an initial questionnaire designed to obtain information about them, their household, and their dog, including: (i) their age and gender, (ii) employment status, (iii) type of housing (e.g., flat, terrace, semi-detached, detached), (iv) household composition (number of adults, number of children), (v) age and gender of dog, (vi) breed of dog, (vii) size of dog, (viii) neutered status, (ix) whether they are the primary carer, (x) whether the dog has been diagnosed with a chronic or terminal illness, (xi) weight of dog (if known), and (xii) self-assessed Body Condition Score (BCS) on a 9-point scale.^4^

##### 3.2.2.2 Health pack

At the end of the initial questionnaire, participants were asked to provide their name and address. We then posted them the health pack designed to help them to manage the weight of their dogs. On receipt of the pack owners were asked to schedule a complimentary online call with a vet (the last author, SM), primarily to check that their dog is healthy and to provide a preliminary evaluation of BCS. However, this call also sought to agree goals for the trial. Specifically, for dogs with a BCS of 6 or above, it was suggested that owners reduce the amount of food they provide by 10% for the duration of the trial (BCIO:007004: Agree behavior goal). Some guidance on the ideal weight for their dog was also provided (BCIO:007006: Agree outcome goal).

##### 3.2.2.3 Follow-up questionnaire

Around ten weeks (*M* = 68 days, *SD* = 7, range: 62 to 92 days) after the pack was posted to owners, they were contacted by email a second time and asked to complete a second questionnaire designed to measure their views of the pack.^5^ On average, participants completed the follow-up questionnaire 85 days after the baseline questionnaire (*SD* = 18, range: 64 to 155 days).

Participants were first asked whether they (and / or members of their household) had used the pack. If so, participants were asked which tools they had used and how. If not, participants were asked why they (or others in their household) had not read or used any parts of the pack. Participants then completed measures of the seven components of acceptability delineated by Sekohn et al. (52): (i) affective attitude, (ii) burden, (iii) perceived effectiveness, (iv) ethicality (the extent to which the intervention has good fit with an individual’s value system), (v) intervention coherence, (vi) opportunity costs, and (vii) self-efficacy. Each component was measured by asking participants to respond to a series of statements on a 5-point scale.

Affective attitude was measured with two items: “*I liked using the health pack*” and “*I enjoyed using the health pack*.” These items proved internally reliable (Cronbach’s alpha = 0.83) and were combined into a single index.

Burden was measured with one item: “*How much effort did it take to use the health pack?*”

Perceived effectiveness was measured with three items: “*Using the health pack helped me to manage my dog’s weight*,” “*The health pack helped me talk to my vet / vet nurse about how to manage my dog’s weight*,” and “*The health pack would support a range of different owners to manage their dog’s weight if needed*.” These items proved internally reliable (Cronbach’s alpha = 0.76) and were combined into a single index.

Ethicality was measured with four items: “*It is okay to put a dog on a diet*,” “*It is wrong to limit the amount of food given to a pet*” (recoded), “*Using the health pack made me feel like I was being kind to my dog*,” and “*Using the health pack made it difficult to show that I love my dog*” (recoded). These items did not prove internally reliable (Cronbach’s alpha = 0.50) and the reliability could not be improved by removing items. Therefore, the items were considered individually in the analyses.

Intervention coherence was measured with one item: “*It was clear to me how using the health pack could help me to manage my dog’s weight*.”

Opportunity costs were measured with two items: “*Using the health pack interfered with my other priorities*” and “*Using the health pack made looking after my dog a chore*.” These items were deemed sufficiently reliable (Cronbach’s alpha = 0.67) to combine into a single index.

Self-efficacy was measured with two items: “*How confident did you feel about using the health pack?*” and “*I found it easy to use the health pack to manage my dog’s weight*.” These items did not prove internally reliable (Cronbach’s alpha = 0.37), which suggested that they reflected slightly different beliefs. Therefore, the items were considered individually in the analyses.

The follow-up questionnaire also asked participants about their behavior (“*Have you (or those in your household) changed the way that you feed, exercise, or interact with your dog over the last 8 weeks?*”). If owners reported changing their behavior, they were asked to specify how their behavior had changed (we categorized owners’ responses as reflecting changes in feeding behavior, exercise behavior and / or interaction with the dog) and whether any changes in behavior were the result of using the health pack (“*Were any of these changes the result of receiving or using the health pack?*”) or something else (“*Please let us know if anything has happened over the last 8 weeks that has affected you or your dog*”). Finally, owners reported whether they had recently weighed their dog (and, if so, what it weighed) and self-assessed their dog’s BCS, again on a 9-point scale.

## 4 Results

All statistical analyses were conducted using SPSS (version 29 for Mac).

### 4.1 Hypothesis 1: owners will be willing and able to self-assess and report their dog’s weight and body condition score (BCS)

Our pre-registered criteria for inferring Hypothesis 1 to be supported was that more than 70% of the owners who completed the follow-up questionnaire would report their dog’s weight and BCS. Of the 91 of the participants who completed the baseline survey, 89 (98%) reported their dog’s BCS and 80 (88%) reported that they knew how much their dog weighed, suggesting that in general, owners were willing and able to self-assess and report their dog’s weight and BCS. On average, the dogs owned by participants who completed the baseline survey were overweight (*Mean* BCS = 5.79, *SD* = 1.11), weighing an average of 19.10kgs (*SD* = 11.44).^6^

### 4.2 Hypothesis 2: owners who are posted a health pack will report using that pack

Our pre-registered criteria for inferring Hypothesis 2 to be supported was that more than 70% of the owners who were posted a health pack report would report using that pack. This hypothesis was supported on the basis that 46 of the 49 participants (94%) who were posted a health pack and completed the follow-up questionnaire reported that they (and / or other members of their household) read or used parts of the pack.

### 4.3 Hypothesis 3: owners will find the health pack acceptable, as evidenced by agreement with items reflecting (i) affective attitudes, (ii) perceived effectiveness, (iii) intervention coherence, (iv) ethicality, and (v) self-efficacy, and disagreement with items reflecting (i) burden and (ii) opportunity costs

Our pre-registered criteria for inferring that owners found the pack acceptable (i.e., support for Hypothesis 3) was that the mean of items reflecting (i) affective attitudes, (ii) perceived effectiveness, (iii) intervention coherence, (iv) ethicality^7^, and (v) self-efficacy would be >3.5 (indicating agreement), while the mean of items reflecting (i) burden, and (ii) opportunity costs would be <2.5 (indicating disagreement). Table 6 shows the descriptive statistics (i.e., mean and standard deviations) for the measures of the acceptability of the health pack.

**Table 6:** Descriptive statistics for measures of the acceptability of the health pack.

Construct	Mean (*SD*)
Affective attitude	3.89 (0.74)
Effectiveness	3.99 (0.68)
Self-efficacy (with respect to using the pack)	4.09 (0.64)
Self-efficacy (with respect to using the pack to manage weight)	4.01 (0.97)
Coherence	4.44 (0.80)
Ethicality	
It is okay to put a dog on a diet	4.66 (0.57)
It is wrong to limit the amount of food given to a pet	1.73 (1.04)
Using the health pack made me feel like I was being kind to my dog	3.98 (0.79)
Using the health pack made it difficult to show that I love my dog	1.66 (0.91)
Burden	2.41 (0.79)
Costs	2.23 (0.99)

SD, standard deviation. Participants were asked to respond to each of the measures of acceptability on a 5-point scale.

There was good evidence that owners found the health pack acceptable, in the sense that, on average, owners liked and enjoyed using the health pack (*M* = 3.89 on a 5-point scale, *SD* = 0.74), believed that it would be effective in helping them and other owners to manage their dogs weight (*M* = 3.99, *SD* = 0.68), and that it was clear how the health pack would do this (*M* = 4.44, *SD* = 0.80). Owners also reported feeling confident using the pack (*M* = 4.09, *SD* = 0.64), that using the pack was relatively easy (*M* = 4.01, *SD* = 0.97), did not require too much effort (*M* = 2.41, *SD* = 0.79) or interfere with their other priorities and / or make looking after their dog feel like a chore (*M* = 2.23, *SD* = 0.99). Owners also found the pack acceptable from an ethical perspective, in the sense that they agreed that it is okay to put a dog on a diet (*M* = 4.66, *SD* = 0.57) and did not feel that using the health pack made it difficult to show love for their dog (*M* = 1.66, *SD* = 0.91).

### 4.4 (exploratory) hypothesis 4: owners who are posted a health pack will report (i) changing the way that they feed, exercise, or interact with their dog over the 8 weeks following receipt of the pack, and (ii) these changes will be the result of receiving or using the health pack

Our pre-registered criteria for inferring Hypothesis 4 to be supported was that more than 70% of the owners who were posted a health pack would report (i) changing the way that they feed, exercise, or interact with their dog over the 8 weeks following receipt of the pack, and (ii) that these changes would result from receiving or using the health pack. 39 of the 49 participants (80%) who were posted a health pack and completed the follow-up questionnaire stated that they (or those in their household) changed the way that they fed, exercised, or interacted with their dog over the last 8 weeks. 36 of these participants (92%) stated that these changes were the result of receiving or using the health pack.

When asked to specify how their behavior had changed, 71% of the participants who were posted a health pack and completed the follow-up questionnaire reported changing the way that they feed (e.g., feeding smaller portions, less treats, or substituting treats for kibble or vegetables), 33% reported changing the amount or way that they exercise (e.g., more walks, throwing a ball in the park), and 51% reported changing the way that they interact with their dog (e.g., playing the games suggested by the Healthy Weight Cards, teaching tricks, or using enrichment toys like puzzles).

### 4.5 (exploratory) hypothesis 5: the weight and body condition score (BCS) of dogs will be lower at follow-up than at baseline

Our pre-registered criteria for inferring Hypothesis 5 to be supported was that the effect size reflecting the effect of time on (i) weight and (ii) BCS would be at least small (i.e., eta (2) > 0.01). We began by evaluating whether the data met the assumptions for the planned repeated measures ANOVA (i.e., no outliers, normally distributed, ratio-level data). Outliers were defined as values +/− 3*SD*s from the sample mean. At baseline, the average weight of the dogs was 19.10 kg (*SD* = 11.44), so we removed dogs weighing in excess of 53.42kgs (one dog: 60 kg^8^) from the estimate of baseline weight. At follow-up, the average weight of the dogs was 17.15 kg (*SD* = 9.81), defining outlying weights as those in excess of 46.58kgs. The heaviest dog at follow-up was reported to be 43.14kgs, so all values at follow-up were retained for analysis. The data on weight was positively skewed (baseline = 1.08, *SE* = 0.40, follow-up = 1.12, *SE* = 0.40) and so a square root transformation was applied prior to analysis to correct the skew (baseline = 0.73, *SE* = 0.38, follow-up = 0.74, *SE* = 0.40).

A repeated measures ANOVA revealed that the average weight of the dogs at baseline (*M* = 17.44, *SD* = 10.24) was higher than that at follow-up (*M* = 17.06, *SD* = 9.44), *F* (1, 33) = 4.30, *p* = 0.046. The effect size for this difference was small (eta (2) = 0.012). The data on BCS was ordinal level and so a non-parametric test was used to evaluate whether there was a difference in BCS between baseline (*M* = 5.70, *SD* = 1.14) and follow-up (*M* = 5.20, *SD* = 0.91).^9^ The Friedman’s test was statistically significant [chi-square (*N* = 47) = 16.67, *p* < 0.001]. The median BCS dropped from 6 at baseline to 5 at follow-up.

## 5 Discussion

The present research drew on behavioral science, insights from veterinary professionals, and feedback from owners to develop a health pack designed to support owners to manage the weight of their companion dogs. The pack targeted determinants of behavior specified by the COM-B model (54), including owners’ psychological capability (e.g., monitoring intake), physical capability (e.g., knowing how to weigh and score BCS), social opportunity (e.g., the behavior of others who may feed the dog), reflective motivation (e.g., awareness of the consequences of dogs being overweight), and automatic motivation (e.g.., pre-existing habits). This was achieved using 21 discrete behavior change techniques^10^ - many of which have been supported by extensive evidence (e.g., monitoring progress (65); action planning (51)), albeit primarily outside the veterinary context. Indeed, although not pre-planned, the health pack included a combination of behavior change techniques (namely, self-monitoring in addition to other techniques derived from control theory (66), such as goal setting) that evidence suggests is particularly effective in promoting healthy eating and physical activity in humans (67). That such findings translate to interventions designed to support dog owners to adopt similar behaviors in relation to their companion animals reiterates the potential of behavioral science in preventive veterinary medicine (22). It also speaks to the importance of using frameworks like the Behavior Change Intervention Ontology (62) to report interventions as so doing can help to build a cumulative evidence base in this context and contribute to broader debates about what behavioral interventions work, when, and for whom (68).

Phase 2 of the research evaluated whether the health pack was likely to be acceptable to owners and thus used, with the resulting changes in behavior. The findings supported this hypothesis. Over 90% of the owners who were posted a health pack and completed the follow-up questionnaire reported that they (and / or other members of their household) read or used parts of the pack. Although we did not measure how often or for how long participants used the materials, this finding is promising. One factor that likely contributed to the relatively high proportion of owners reporting using the pack is that owners found the health pack acceptable. That is, we found that owners typically liked and enjoyed using the health pack, believed that it would be effective in helping them and other owners to manage their dog’s weight (including talking to veterinary professionals about managing weight), and that it was clear how the health pack would do this. Owners also reported feeling confident using the pack, that using the pack was relatively easy and did not interfere with their other priorities and / or made looking after their dog feel like a chore. The finding that the pack was acceptable to owners is reassuring and, taken together with evidence that owners engaged with the materials, suggests that a printed health pack is a suitable way to deliver an intervention designed to support dog owners to manage the weight of their companion animals.

Although the primary purpose of the research reported in this paper was to evaluate the acceptability of the materials, we also explored potential effects on owners’ behavior and outcomes for their companion animals. The findings were encouraging – 80% of the owners who were posted a health pack and completed the follow-up questionnaire stated that they (or those in their household) changed the way that they fed, exercised, or interacted with their dog over the last 8 weeks - and the majority (92%) stated that these changes were the result of receiving or using the health pack. Notwithstanding some limitations (e.g., that the analyses are based on participants who completed the follow-up questionnaire – discussed further below), these are encouraging levels of compliance and stand in contrast with the relatively low levels of compliance reported with respect to other interventions. For example, evidence that just 21% of owners follow recommended therapeutic diets (38) and just 53% of owners follow physical activity recommendations (39). Although we did not expect to observe changes in weight and BCS (the study was not powered to detect such differences and the follow-up period was relatively short), there were indications that the weight and BCS of the dogs in the study improved. These findings need to be taken in the context of several limitations (below); however, they provide the basis for a full RCT to formally assess the effectiveness of the health pack against a control condition. Our suggestion would be that this trial measure continued as well as initial use of the pack and independently assess weight and BCS at a series of appropriate follow-up points (e.g., 3 and 6 months). The control condition could be standard veterinary advice (e.g., if the health pack was used to augment a consultation or nurse-led clinic).

### 5.1 Limitations

The findings of the present research need to be considered in the context of some limitations. First, 29 of the 78 participants (37%) who were posted a health pack did not complete the follow-up questionnaire. We tested our hypotheses by considering the responses of participants who completed the questionnaires; however, an alternative, more conservative test of Hypotheses 2 and 4 would be to assume that participants who did not complete the follow-up questionnaire did not use the pack (Hypothesis 2) or change the way that they feed, exercise, or interact with their dog (Hypothesis 4). Under this assumption (akin to an intention-to-treat analysis), neither hypothesis would be supported (i.e., 59% of owners who were posted a health pack report reported using the pack and 50% reported changing their behavior) and rates of compliance would be closer to estimates from other evaluations of interventions outside the laboratory (40). Attrition is a common problem in trials of interventions, particularly those delivered remotely (69) and there is debate as to how to handle missing data (70). However, future research should consider (i) further efforts to recontact participants who do not respond to identify if this is because (a) they have not used or liked the pack or (b) simply have not completed questionnaire (e.g., because they have not had time or have forgotten), and (ii) how to promote engagement and reduce attrition.

A second limitation of the present research was that weight and BCS were reported by owners rather than independently verified. Evidence suggests that owners’ typically underestimate their dogs’ BCS and weight (17, 18, 20). However, owner’s assessments are correlated with veterinary professionals’ assessments of BCS (*r* = 0.58 (20)) and the aim of our research was to evaluate change in weight from pre- to post-intervention. Therefore, although the present research may underestimate absolute levels of BCS, there is no reason to think that BCS would be underestimated to a greater extent at follow-up (indeed, given the instructions that owners received around assessing BCS in the health pack, we might expect the follow-up assessments to be more accurate and thus higher). Therefore, the estimation of change is likely more reliable than the absolute BCSs. Having said this, if the health pack were to be evaluated in a formal RCT with BCS as the primary outcome, it would be helpful to include more objective assessments, either directly by a veterinary professional or remotely. For example, Gant et al. (71) and Webb et al. (20) both found that it is possible to assess BCS from pictures that owners submit, albeit with the caveat that some owners struggle to take and submit appropriate pictures.

## 6 Conclusion

Overweight and obesity is a severe and prevalent problem among companion animals and there is a need for interventions that can support owners to make changes to their behavior. The present research shows that it is possible to develop materials to support owners to manage the weight of their companion animals that are likely to be used and may positively affect outcomes. The findings thus provide the basis for a full RCT to formally assess the effectiveness of the health pack against a control condition.

In addition to more formal evaluation of the effect of the pack on outcomes, future research could also consider how the pack might be used. The tools were designed to be used by owners independently of veterinary professionals. However, it may be necessary to check that it is appropriate to limit food intake (e.g., via a brief – remote if needed – consult to check the health status of the dog, as in the present research) prior to owners doing so. If so, future research could also evaluate potential ways to provide this check – e.g., online decision tools supported by AI, in person or remote consult, with a vet nurse or trained staff at, for example, a pet store. Alternatively, or in addition, the pack might be used to support veterinary professionals to work with owners. For example, the pack could provide resources for owners to take home after a veterinary consultation. This approach would fit with owner’s feedback on the initial set of tools, which suggested that some degree of personalisation would be helpful to ensure that the tools work for different dogs and households. Owners also felt that the pack would help them to discuss weight management with professionals. In this sense, the tools might facilitate conversations around weight that many veterinarians find difficult (32).

## Data Availability

The datasets presented in this study can be found in online repositories. The names of the repository/repositories and accession number(s) can be found below: Open Science Framework: https://doi.org/10.17605/OSF.IO/D9ZTP.

## Footnotes

^1^Receptionists were included because, although not trained in veterinary medicine, they often have good relationships with patients and the time spent talking may help to identify some of the more everyday experiences of dog owners.

^2^Potential participants were provided with a BCS chart to facilitate this assessment and were also asked to submit a photo of their dog to allow some initial verification by the research team.

^3^We recognise that weight in itself is not a measure of overweight / obesity – hence the inclusion of BCS. However, owners needed to be willing and able to weigh their dogs to benefit from some tools in the health pack (e.g., the weight loss progress chart) and this data allowed us to evaluate changes in weight as a secondary outcome (Hypothesis 5).

^4^Participants were also asked to complete measures of beliefs specified by Protection Motivation Theory (64) using items from our previous research (20). This included measures of (i) threat appraisal – severity (“Overweight and obesity cause severe problems in dogs” and “The health risks associated with overweight and obesity in dogs are severe”), (ii) threat appraisal – vulnerability (“The chances are high that my dog is, or will become, overweight” and “My dog easily puts on weight”), (iii) coping appraisal - response efficacy (“Ensuring my dog is the correct weight will help to reduce health problems” and “Ensuring my dog is the correct weight will mean that they have a long and healthy life”), and (iv) coping appraisal - self-efficacy (“I am capable of keeping my dog at a healthy weight” and “I am capable of helping my dog to lose weight if needed”). These items were not part of the pre-registered analyses and so are not reported further.

^5^Our pre-registered aim was to recontact owners eight weeks (56 days) after posting the pack, but a number of logistical challenges, including the Christmas vacation meant that owners were recontacted slightly later than planned.

^6^This estimate excludes one outlying weight (60 kg) as described in the analysis of Hypothesis 5. We also removed the weight data for one participant as the increase in weight was improbable (a chihuahua which was reported to weigh 4 kg at baseline, but 20 kg at follow-up).

^7^Note that this hypothesis was pre-registered as predicting *disagreement* with items reflecting ethicality. However, the questions were coded such that high scores indicated that owners viewed the health pack as a good fit with their value system (e.g., that they agreed that “it is okay to put a dog on a diet”) and so agreement with these items indicated that the health pack was acceptable to owners.

^8^The owner did not report the weight of this dog at follow-up.

^9^Note that the use of a non-parametric test represents a deviation from the pre-registration which specified that a second repeated measures repeated measures ANOVA would be used to examine changes in BCS as a function of time. However, the planned analysis neglected to account for the fact that BCS is an ordinal-level variable.

^10^Some behavior change techniques were delivered by more than one component of the intervention. For example, both the weight loss progress chart and monthly milestones chart prompted owners to self-monitor the outcomes of their behavior (BCIO:007025).
