# Supplementary material for: Developing and evaluating a health pack to support dog owners to manage the weight of their companion animals
Source: Front Vet Sci. 2025 Jan 7;11:1483130. doi: 10.3389/fvets.2024.1483130 (PMC11746120; doi:10.3389/fvets.2024.1483130)
Supplement: Supplementary file 1 [file Table_1.DOCX]

Supplementary Material

# Interview guide

1. **Welcome and introduction**
   - Introduce self. Independent Research Company.
   - Follow the MRS Code of Conduct – assures anonymity.
   - How the information will be used. Ultimately to develop and improve health information and support for owners of dogs who are overweight
   - Discussion open and informal, there are no right or wrong answers, we are simply interested in hearing your opinions.
   - There won’t be any trick questions. It will be an informal chat and this guide will be used as just that – a guide to keep us on track
   - Length of session
   - Participant has the right to not answer questions they would rather not, has the right to end the discussion at any point and has the right to request that information given not be used
   - Audio recording. Permissions. Explain that audio will be securely handled, secured and deleted at end of the project.
   - Check that the participant is happy to continue.
2. **Personal introductions (5 Minutes)**
   - - About you
       - Family, homelife, pets
     - What is your profession?
       - Veterinarian
       - Veterinary Nurse
       - Veterinary Receptionist
     - How long have you been a Veterinarian/Veterinary Nurse/Veterinary Receptionist?
     - How long have you been qualified?
     - How long have you been at your current position?
     - Where else have you worked?
     - Can you tell me about the practice you work at?
       - Where is it?
       - Who else works there?
       - How many members of staff?
       - How many clients?
       - What are some of your favourite things about your job?
       - And your least favourite?
3. **Experience of dog obesity (5 minutes)**
   - What experience do you have with overweight dogs?
     - - How common is it at your practice?
       - What are the different situations you see?
       - Are the breeds that you notice it to be more common with?
   - Do you have any current clients with overweight dogs?
     - - How do these clients present to you? Do any clients come and see you specifically about the problem? Tell me about the typical journey for your clients in acknowledging their dog is overweight
     - What are the common *themes* you see in owners with overweight dogs?
     - What issues do they face when it comes to weight management of their dog?
       - Can you give me some examples of the different cases you come across – **Moderator probe fully**
       - And what would you say are the main common *challenges* that owners have?
     - What is their understanding of obesity in dogs?
     - How do they manage these issues currently?
4. **What materials or information are available at your practice to support dog owners to manage the weight of their dogs? (5 minutes)**
   - - How do you give this information?
     - When do you give them the information? All at once or at different points in the weight loss journey?
     - What support is available for dog owners outside of your practice currently?
       - What works well?
       - What’s missing?
     - Do you think that owners are aware of what information is available outside of your practice?
     - What information/support do you see as being the most helpful? And the least?
     - Do you have any understanding on how they are used by the owners? What are their impacts on pet owners’ behaviours?
5. ***Moderator read out:*** *We are specifically interested in how owners can be supported to change their behaviour in a way that will help their dogs to lose weight and/or maintain a healthy weight. The main aim is to translate into practice some of the practical recommendations for owners on weight management to integrate them more easily in their daily life*

*You may have heard of the COM-B model? The COM-B model proposes that behaviour occurs when people are (i) motivated, (ii) capable, and (iii) have an appropriate opportunity. In turn, the implication is that problems with behaviour (e.g., not acting or acting inappropriately) may reflect a problem with motivation, capability (which refers to whether the person has the knowledge, skills and stamina needed to perform the behaviour), and / or opportunity (which refers to whether the physical and social environment support the behaviour).*

*So a person who feeds too many treats to their dog may do so because they are not motivated to do otherwise (e.g., they don’t think that obesity is a problem), because they are not capable of doing otherwise (e.g., they use treats to control the dog and don’t have other ways of doing so), and / or a problem of opportunity (the treats are in a bowl on the side, so the owner tends to just give the dog a treat as they walk past).*

*The idea is that if veterinarians can work with owners to identify why they behave the way that they do then they can help them to identify strategies for changing these things (e.g., increasing motivation, changing the social or physical environment).*

*MODERATOR: IF NEEDED, SHOW PARTICIPANT THE COM-B MODEL SIMPLE VERSION AND EXPLAIN THE SEGMENTS.*

*With this in mind, I am now going to ask you some questions about your clients that will help us to see how they fit on the COM-B model.* (10 mins)

- When you think about your clients and the challenges that we discussed earlier on, how do you feel motivation plays as part in the challenge? Are there cases of positive motivation you can think of? Any negative motivation cases? **Moderator: probe fully before moving on** **and use the COM-B model to refer to if required**
- When you think about your clients and their overweight dogs, how do you feel capability plays as part in the behaviour? Are there cases of positive capability you can think of? Any negative capability cases? **Moderator: probe fully before moving on** **and use the COM-B model to refer to if required**
- When you think about your clients and their overweight dogs, how do you feel opportunity plays as part in the behaviour? Are there cases of positive opportunity you can think of? Any negative opportunity cases? **Moderator: probe fully before moving on** **and use the COM-B model to refer to if required**

1. I’m now going to show you some concepts for a potential new health information pack for owners of overweight dogs and I would like you to consider how these ideas might work to help positively change behaviour in owners. **(15 minutes)**

**Moderator: present each proposition/concept in turn and ask following questions for each:**

- - What are your initial thoughts?
  - What do you understand by this idea?
  - Is there anything you don’t understand/isn’t clear
  - Thinking about the dog owners you work with, what do you think would/would not work well, if anything
  - How do you see yourself using it to support dog owners in your working life?
  - At what point in the weight loss journey do you envisage this tool being used?
  - Who do you think would use it? **Moderator:** **Probe on types of dogs, breeds, owners, challenges**

1. Now that we have gone through the different ideas **(5 minutes)**:
   - What are your overall thoughts on the combination of tools here?
   - Building on what we previously discussed what would be for you the missing tricks/tips you are using that could be integrated in this package
   - Once you have handed this ‘toolkit’ to the owner, what would you envisage to be the journey from there in terms of your continued support/contact?
2. And finally, is there anything else you would like to add? What is your main takeaway from our discussion today? What one piece of advice would you give to ensure that this health pack is the best it can be?

**Moderator read out: thank you very much for all your input today. Your responses will be really helpful in creating a successful health pack for dog owners with overweight dogs.**

Thank, close and arrange payment of incentive/charity donation.
